# Begomovirus Transmission to Tomato Plants Is Not Hampered by Plant Defenses Induced by *Dicyphus hesperus* Knight

**DOI:** 10.3390/v16040587

**Published:** 2024-04-10

**Authors:** Saioa Legarrea, Angela Gabrielle LaTora, Alvin M. Simmons, Rajagopalbabu Srinivasan

**Affiliations:** 1 Department of Entomology, University of Georgia, 1109 Experiment Street, Griffin, GA 30223, USA; gabrielle.latora@uga.edu; 2 Department of Food and Agriculture, University of La Rioja, C/Madre de Dios, 53, 26006 Logroño, Spain; 3 University of Georgia Extension Fulton County, 7741 Roswell Road NE, Room 248, Sandy Springs, GA 30350, USA; 4 U.S.D.A.—Agricultural Research Service, U.S. Vegetable Laboratory, 2700 Savannah Highway, Charleston, SC 29414, USA; alvin.simmons@usda.gov

**Keywords:** plant defenses, plant–virus–vector interactions, *Bemisia tabaci*, TYLCV, mirid, begomovirus, jasmonic acid, salicylic acid

## Abstract

Plants can respond to insect infestation and virus infection by inducing plant defenses, generally mediated by phytohormones. Moreover, plant defenses alter host quality for insect vectors with consequences for the spread of viruses. In agricultural settings, other organisms commonly interact with plants, thereby inducing plant defenses that could affect plant–virus–vector interactions. For example, plant defenses induced by omnivorous insects can modulate insect behavior. This study focused on tomato yellow leaf curl virus (TYLCV), a plant virus of the family *Geminiviridae* and genus *Begomovirus*. It is transmitted in a persistent circulative manner by the whitefly *Bemisia tabaci* Gennadius (Hemiptera: Aleyrodidae), posing a global threat to tomato production. Mirids (Hemiptera: Miridae) are effective biological control agents of *B. tabaci*, but there is a possibility that their omnivorous nature could also interfere with the process of virus transmission. To test this hypothesis, this study first addressed to what extent the mirid bug *Dicyphus hesperus* Knight induces plant defenses in tomato. Subsequently, the impact of this plant–omnivore interaction on the transmission of TYLCV was evaluated. Controlled cage experiments were performed in a greenhouse setting to evaluate the impact of mirids on virus transmission and vector acquisition by *B. tabaci.* While we observed a reduced number of whiteflies settling on plants exposed to *D. hesperus*, the plant defenses induced by the mirid bug did not affect TYLCV transmission and accumulation. Additionally, whiteflies were able to acquire comparable amounts of TYLCV on mirid-exposed plants and control plants. Overall, the induction of plant defenses by *D. hesperus* did not influence TYLCV transmission by whiteflies on tomato.

## 1. Introduction

Plants respond to biotic attack by inducing plant defenses mediated by phytohormones that act as central regulators of the response [1,2,3]. Ultimately, the activation of defensive routes leads to the production of enzymes, toxins, or volatile compounds that aim at reducing the impact of the attacking organism [1,4]. For example, when a plant is attacked by a chewing caterpillar, it activates the route of the jasmonic acid pathway (JA), leading to the production of JA-inducible proteins (JIP). Once in the midgut, such proteins disrupt digestion, thereby hampering herbivore performance [5,6]. Other organisms, such as biotrophic fungi [2] and certain plant viruses [3], activate the route of the salicylic acid pathway (SA) that can translate into a local Hypersensitive Response (HR) or even trigger Systemic Acquired Resistance (SAR) [7,8]. In addition, the SA and JA defensive pathways often induce the production of volatile compounds (e.g., Terpenes and MeSA), which can cascade to other organisms [1]. For example, volatiles can serve as cues to attract natural enemies that feed on herbivores [4,9]. Interestingly, plant defenses can have impacts that go far beyond the organism that causes the attack.

The induction of plant defenses can result in increased resistance, and this is proposed as a novel strategy to control plant pathogens, including viruses [10,11]. A paramount example is the use of SA defense elicitors that can suppress symptom expression and delay or reduce virus infections [12,13,14], along with suppression of other plant pathogens in multiple crops [8]. Other defense elicitors such as Green Leaf Volatiles (GLVs) have been shown to reduce virus transmission in tomato via a strong defense response on the JA pathway that interfered with whitefly inoculation [15]. Plant defenses could also be elicited by other organisms that serve as biocontrol agents, such as mirids. Mirids are omnivorous insects that exert a dual role in the control of pests and pathogens [16]. First, mirids directly feed on herbivores, such as whiteflies, thereby reducing vector densities [17,18]. Secondly, as omnivores, when mirids feed on plants, they induce plant defenses that can affect herbivore performance and pathogen accumulation [19,20,21]. Plant defenses induced by mirids are activated via the JA, SA, or abscisic acid (ABA) pathway; however, the induction is species-specific [16,20,22], and consequently, the impact on the plant is also variable depending on the insect species [22]. Despite such differences, defense induction by mirids was shown to negatively affect the performance of a variety of herbivores (e.g., spider mites, whiteflies, and thrips) via antibiosis or antixenosis mechanisms [20,22,23,24]. Overall, mirids and the plant defense compounds that are elicited by them are proposed as effective pest management strategies for multiple greenhouse pests [23,25].

Tomato yellow leaf curl virus (TYLCV) is a plant virus of the family *Geminiviridae* and genus *Begomovirus*. It is among the 13 causal agents of tomato yellow leaf curl disease (TYLCD) that affect tomato crops globally [26]. Symptomatic plants show curling and yellowing of the youngest leaves, while the most devastating effect is stunting, which can drastically limit tomato yields [27]. TYLCV infection in plants induces plant defenses in the SA pathway [28]. Interestingly, the induction of plant defenses by TYLCV favors vector performance by exploiting the JA-SA antagonism [28]. The main vector of TYLCV is *Bemisia tabaci* Gennadius, a species complex that transmits TYLCV in a persistent manner. *B. tabaci* B cryptic species (formerly known as B biotype, Middle East Asia Minor 1 (MEAM1)) is considered the most efficient vector in the USA, and management strategies against the virus are often directed towards reducing vector densities and the use of resistant plants that accumulate lower levels of TYLCV [27]. Both TYLCV and *B. tabaci* have been shown to respond negatively to the induction of plant defenses in tomato by defense elicitors [15]. However, it remains unknown whether the induction of plant defenses by mirids in tomato could result in increased resistance against the transmission of begomoviruses.

Plant defenses are central to the myriad of interactions that plants establish with other organisms [1], and the induction of plant defenses is emerging as a component of integrated pathogen management [10]. These defenses can be induced by biocontrol organisms such as mirids [16,23]. Here, we hypothesize that the defenses induced by the mirid bug *Dicyphus hesperus* Knight could influence the transmission and accumulation of TYLCV by its whitefly vector. To test this hypothesis, three complementary experiments were conducted under greenhouse cage conditions to evaluate the following: (1) the extent to which *D. hesperus* induces plant defenses in tomato plants in a local and a distal leaf; (2) whether plants exposed to mirids would modulate the transmission of TYLCV by viruliferous vectors; and (3) whether exposure to mirids may influence the acquisition of TYLCV by *B. tabaci.* In these experimental settings, *D. hesperus* induced plant defenses, but this did not affect virus transmission. While mirid bugs can contribute to the management of relevant insects and the viruses transmitted by them, we suggest that a deep understanding of the particularities of each system is required, as the effects may be species-specific and dependent on the molecular mechanisms that are in place.

## 2. Materials and Methods

### 2.1. Plants, Virus, and Insects

Tomato plants *Solanum lycopersicum* L. cv. Florida 47 F1 (Holmes Seed Co., Canton, OH, USA) were used in this study and grown in the greenhouse for 4–6 weeks. Plants were established from seeds and transplanted to 10 cm diameter pots with a plant-growing substrate (Promix; Premier Horticulture Inc., Quakertown, PA, USA). Plants were fertilized weekly with Miracle-GRO (Scotts Miracle-Gro products, Inc., Marysville, OH, USA) at a concentration of 1 g/L. Prior to the experiments, plants were transplanted to 1 L pots. *B. tabaci* B cryptic species was first collected in Tifton, GA, USA, in 2009 [27]. The colony was confirmed to belong to the B cryptic species by sequencing the cytochrome c oxidase subunit I (COI) gene (GenBank accession number: MN970031). The colony was maintained virus-free on cotton plants (*Gossypium hirsutum* L.) inside insect-proof cages in a greenhouse. The TYLCV-IL isolate (GenBank accession number: KY965880) was originally collected in an infected tomato field in 2009 [27]. TYLCV-IL was maintained via serial whitefly inoculations on tomato using clip cages on 4-week-old plants. Plants and whiteflies were maintained inside insect-proof cages (BugDorm, 45L × 45W × 90H cm Megaview Science Co., Taichung, Taiwan) in a greenhouse under controlled temperature (26–29 °C) and photoperiod (15 h:9 h; L:D). Viruliferous whiteflies were obtained by releasing adults inside a cage with TYLCV-symptomatic tomato plants during an acquisition access period (AAP) of 72 h.

The mirid species used in this study, *D. hesperus*, is naturally distributed in the area of study (the USA), and it is a predator against whiteflies [18]. It was chosen for these experiments for that reason, and the initial colony was provided by IPM Laboratories, Inc. (Locke, NY, USA). Mirids were maintained inside ‘bugdorm’ cages (60L × 60W × 60H cm Megaview Science Co., Taichung, Taiwan) in a climate box with controlled temperature (25 °C), 60% RH, and photoperiod conditions (14 h:10 h; L:D). To maintain the colony, mullein (*Verbascum thapsus* L.) plants were used as a plant substrate, and Entofood (*Ephestia kuehniella* eggs + *Artemia* spp. cysts, Koppert Biological Systems, Inc., Howell, MI, USA) was provided as diet supplement ad libitum twice per week.

### 2.2. Plant Defenses Induced by Mirids in Plants without TYLCV

To assess the induction of plant defenses by mirids, tomato plants were grown for 6 weeks. Subsequently, 10 adult mirid bugs (5 males and 5 females) were confined to leaf number 4 (counting from the base of the plant) by using an insect-exclusion polyester bag tightened to the petiole with an elastic band following the methods established in Zhang et al. [20]. An insect-exclusion bag without mirids was also applied to control plants. Plants were maintained in insect-exclusion cages in the greenhouse as described above. After 4 days, plant material was collected from the local leaf exposed to the mirid bugs (leaf #4). To evaluate the systemic induction of plant defenses, plant material was also collected from a distal point; the youngest expanded leaf of the plant (leaf #6 to #8 counted from the base of the plant). From each leaf, 100 mg of plant material was flash frozen in liquid N_2_ and stored at −80 °C in a freezer for further analysis. For each experiment, 5 plants were exposed to mirids, and 5 plants served as control plants. The experiment was conducted three times for a total number of 12–15 replicates.

Total RNA from plant samples was isolated using an RNA extraction kit (Spectrum^TM^ Plant Total RNA kit, Sigma-Aldrich Co., Saint Louis, MO, USA). RNA working concentration was adjusted to 500 ng/μL, and a DNAse I treatment (DNAse I Amplification grade, Sigma-Aldrich Co., Saint Louis, MO, USA) was performed prior to cDNA synthesis using Oligo dT (GoScript^TM^ Reverse Transcriptase, Promega, Madison, WI, USA) following manufacturer instructions. The gene expression of defense marker genes was conducted as described in Alba et al. [29]. In short, Sybr Green PCR (Gotaq qPCR Master mix, Promega, Madison, WI, USA) was performed in a real-time thermo-cycler (QuantStudio 3, Applied Biosystems, Thermo Fisher Scientific, Waltham, MA, USA) to amplify the cDNA of the gene of interest (Table 1). The amplification of a reference gene (Actin or EF1) was also performed in the same reaction plate. Expression data were shown in relation to the reference gene of interest by applying the 2^–ΔΔCT^ method [30].

### 2.3. Virus Transmission by B. tabaci in Plants Exposed to Mirids

To assess the impact of mirid bugs on the transmission of TYLCV, 6-week-old tomato plants were first exposed to mirids as explained above. Ten adults (5 males and 5 females) were confined to leaf #3 using an insect-exclusion bag attached to the petiole. The bag was attached to control plants without mirids. Groups of 6 plants were arranged in a circle inside three 1 m^3^ BugDorm cages (Megaview Science Co., Taichung, Taiwan) in the greenhouse. One cage consisted of 6 non-exposed control plants that served as a comparison for a second cage that had six plants with mirid-exposed plants. In the third cage, a choice experiment was devised as three plants exposed to mirids were alternated with three control plants. Mirids were allowed to puncture the plants for four days, presumably inducing plant defenses. Then, 50 viruliferous adult whiteflies were released in the center of each cage and allowed to settle on the plants. After 3 days, whiteflies were recaptured using an aspirator and counted on each plant. Three weeks after whitefly inoculation, the number of symptomatic plants was evaluated. TYLCV accumulation in plants was assessed 3 weeks after whitefly release by collecting 100 mg of plant material from the youngest expanded leaf and stored at −80 °C freezer for further analysis. The experiment was conducted three times, altering the position of the cages in the greenhouse to exclude any position bias in the choice experiment. DNA was extracted from the plant sample (GenJET Plant Genomic DNA Purification kit, Thermo Fisher Scientific, Waltham, MA, USA), and the absolute quantification of TYLCV by qPCR was performed as in Legarrea et al. [37]. Briefly, Sybr Green PCR (Gotaq qPCR Master mix, Promega, Madison, WI, USA) was performed. Primers to quantify TYLCV amplified a sequence of 102 bp in the C2 gene [TYLC-C2-For (5′-3′): GCAGTGATGAGTTCCCCTGT and TYLC-C2-Rev (5′-3′): CCAATAAGGCGTAAGCGTGT]. Absolute quantification was performed with a standard curve consisting of six 10-fold dilutions starting at a concentration of 2 × 10^9^ copies of a plasmid [pJET1.2 containing the sequence of interest and provided by GenScript USA, Inc (Piscataway, NJ, USA)], and standard samples were run in triplicates. Two technical replicates were performed per sample, and reactions without plant DNA served as negative controls. The reaction was conducted in the same thermo-cycler as above under standard conditions [an initial denaturation step (3 min at 95 °C), followed by 40 cycles of denaturation (15 s at 95 °C), and a combined step of annealing and extension (60 °C for 60 s), and followed by a melting curve analysis to evaluate the specificity of fluorescence signal].

### 2.4. Virus Acquisition by Whiteflies from TYLCV-Infected Plants Exposed to Mirids

For this experimental set-up, TYLCV symptomatic plants were chosen and exposed to one pair of mirid bugs inside clip cages. Control plants were also TYLCV-symptomatic but held a clip cage without mirids. After a period of four days, the same tomato leaf was exposed to whiteflies for virus acquisition as described in Legarrea et al. [37]. The whiteflies were introduced in the same or a different clip cage, resulting in three treatment combinations: (1) Mirid + Whitefly treatment: whiteflies and mirids interacting with the plants in different clip cages; (2) Mirid treatment: whiteflies and mirids interacting in the same clip cage; and (3) Control: plants not exposed to mirids. After three days, the whiteflies were transferred to cotton plants to clear the gut contents as described in Legarrea et al. [37]. Pools of 5 whiteflies were collected, frozen, and processed to extract DNA (DNeasy Blood and Tissue kit, Qiagen, Valencia, CA, USA), and TYLCV accumulation was estimated as described above.

### 2.5. Data Analysis

Data analysis was conducted using SPSS IBM v.24. The expression of marker genes was first log-transformed to achieve normality and then subjected to a Generalized Linear Models (GLM), with identity link function and a normal distribution. The combination of exposure to mirids and position on the plant was set as a fixed factor. Pairwise comparisons following Bonferroni tests were used to separate significantly different groups (α = 0.05). Fisher’s exact test was used to determine if the success of virus inoculation (measured as the presence or absence of symptoms in the plant) was associated with the exposure of the plants to mirids. Moreover, the accumulation of TYLCV in the plant tissue or in the whiteflies was separately analyzed for choice and non-choice settings. For each setting, TYLCV accumulation was first checked for normality of data distribution and subsequently compared between plants exposed to mirids and control plants using *t*-test (α = 0.05). A GLM with a Poisson distribution and a log link function was applied to the number of whiteflies recaptured on the plants. ‘Exposure to mirids’ was set as a fixed factor, and the dataset was analyzed separately for choice and non-choice settings. The accumulation of TYLCV in the whiteflies was first log-transformed to achieve normality and then subjected to GLM with identity link function and normal distribution as above, in which ‘treatment combinations’ was set as a fixed factor.

## 3. Results

### 3.1. Tomato Plant Defenses Induced by D. hesperus

**Figure 1:**
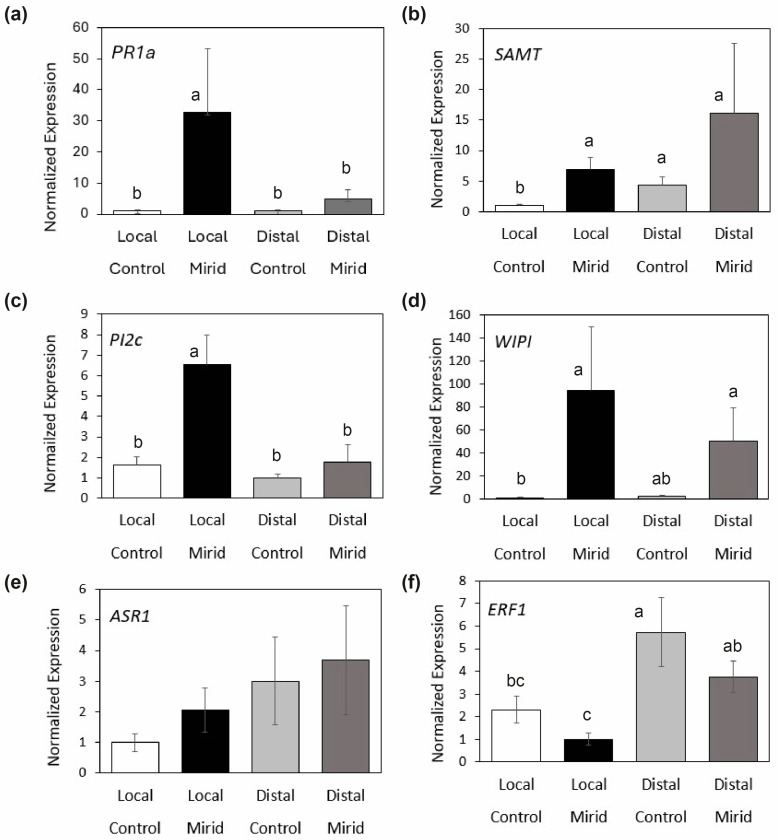
Relative expression of marker genes in the (**a**,**b**) salicylic acid (*PR1a*; *SAMT*); (**c**,**d**) jasmonic acid (*PI2c*; *WIPI*); (**e**) abscisic acid (*ASR*); and (**f**) ethylene (*ERF*) pathways was performed following the delta-delta Ct method with actin or EF as reference genes. Different letters above bars indicate significant differences after GLM and Bonferroni comparisons (α = 0.05) on log-transformed data. Bars show means ± SEM, and they are scaled to the lowest average value of normalized expression.

The mirid bug *D. hesperus* induced plant defenses in tomato plants after an exposure period of four days. Both marker genes in the salicylic acid pathway changed by exposing the plants to mirids. The expression of *Pathogenesis-related protein 1a* (*PR1a*), a common marker gene of the salicylic acid pathway, was induced by mirids, but exclusively on the local leaf that was exposed to mirids (GLM, Chi^2^ = 25.87, df = 3, *p* < 0.0001, Figure 1a). In contrast, *Salicylic acid carboxyl methyltransferase* (*SAMT*) was not only induced locally by mirids, but also a higher expression was found at the distal leaves, regardless of mirid exposure (GLM, Chi^2^ = 32.355, df = 3, *p* < 0.0001, Figure 1b).

The two marker genes in the jasmonic acid pathway were induced by mirids [*Proteinase Inhibitor IIc* (*PI2c*): GLM, Chi^2^ = 27.391, df = 3, *p* < 0.0001; *Wound-induced Proteinase Inhibitor II* (*WIPI*)*:* GLM, Chi^2^ = 10.886, df = 3, *p* = 0.012]. The strongest plant response to mirids was observed at the local leaf by a four-fold induction of *PI2c* compared with the control leaves (Figure 1c). The expression of *WIPI* was higher in plants exposed to mirids at both the local and distal leaves, and this was significantly different from the control local leaf (Figure 1d).

The expression of a marker gene in the abscisic acid pathway [*Abscisic acid Stress Ripening protein 1* (*ASR1*)] remained unaltered in mirid exposed plants compared with the controls (GLM, Chi^2^ = 3.773, df = 3, *p* = 0.287; Figure 1e). The expression of the marker gene *Ethylene responsive factor (ERF)* was different between treatment combinations (GLM, Chi^2^ = 31.43, df = 3, *p* < 0.0001). The gene expression of *ERF* was lower on the leaf exposed to mirids when compared with the control distal leaf, whereas the level of expression in the other samples fell in between (Figure 1f).

### 3.2. Virus Transmission in Plants Exposed to D. hesperus

**Figure 2:**
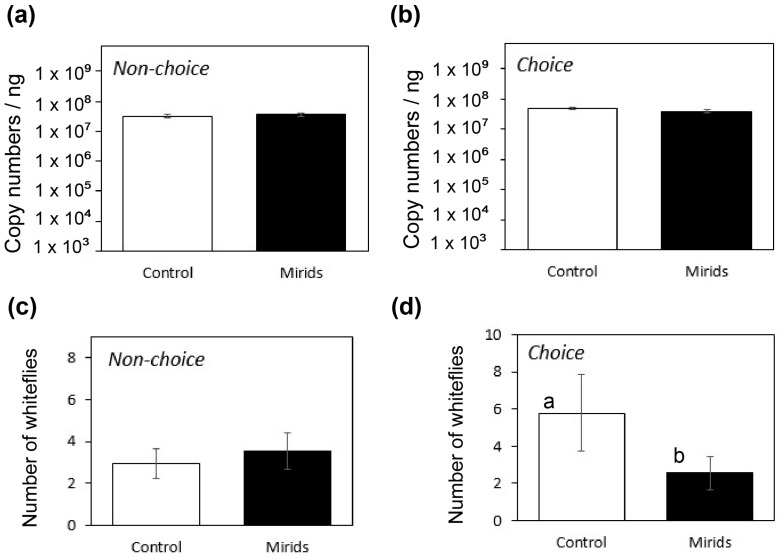
Influence of mirids on virus transmission and whitefly settling. Panels (**a**,**b**) show TYLCV accumulation (mean ± SEM) on tomato leaves in a non-choice and a choice greenhouse set-up, respectively. The number of viruliferous whiteflies recaptured in the plants (mean ± SEM) is shown for (**c**) non-choice and (**d**) choice set-ups under greenhouse conditions. Different letters indicate significant differences in the number of whiteflies recaptured on mirid-exposed and control plants (Poisson distribution, log-link function).

The percentage of plants showing symptoms three weeks post inoculation was 89% (24/27) for plants exposed to mirids and 85% (23/27) for control plants. Exact Fisher test indicated that TYLCV infection was not significantly associated with the exposure to mirids (*p* = 1.000). Molecular analysis showed that TYLCV was detected in all inoculated plants, reaching a 100% transmission success regardless of the presence of mirids. In addition, qPCR quantitation showed that virus accumulation in the plant tissue was not significantly different between tomato plants exposed to mirids and control plants (Figure 2a,b). This result was consistent whether the experiment was conducted in a choice (*t* = −0.79, df = 34, *p* = 0.43) or in a non-choice setting mode (*t* = −0.77, df = 16, *p* = 0.45). The accumulation of TYLCV in the leaf was in all cases in the range of 10^7^ copies per nanogram of leaf tissue.

Viruliferous whiteflies in this study were recaptured after an inoculation access period (IAP) of three days. The numbers of viruliferous whiteflies recaptured per plant did not differ between mirid-exposed and control plants in non-choice settings (GLM, Chi^2^ = 1.031, df = 1, *p* = 0.309, Figure 2c). However, when whiteflies were given a choice by distributing mirid-exposed and control plants in the same cage, a higher number of viruliferous whiteflies was recaptured on control plants (GLM, Chi^2^ = 11.511, df = 1, *p* = 0.001, Figure 2d).

### 3.3. Acquisition by Whiteflies of TYLCV from Plants Exposed to D. hesperus

**Figure 3:**
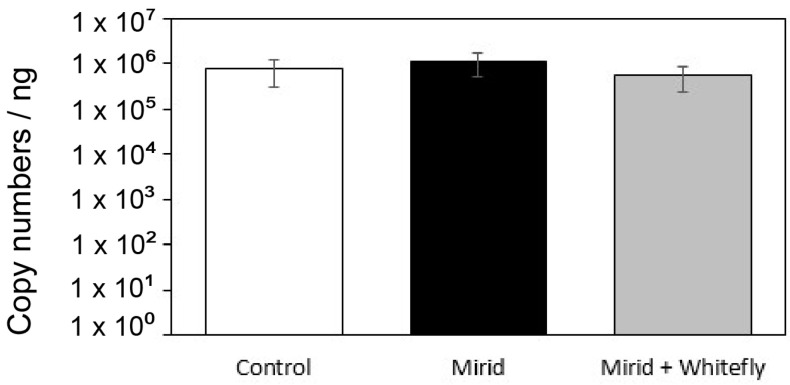
Influence of mirids on virus acquisition by the whitefly (*B. tabaci*). TYLCV copy numbers (mean ± SEM) accumulated on whiteflies after an acquisition access period of 72 h in clip cages. Clip cages were attached to TYLCV symptomatic leaves, and three treatments were assayed, i.e., (1) Control (i.e., plants without exposure to mirids; white bar); (2) Mirid treatment (i.e., plant exposed to mirids in the same clip cage as the whiteflies; black bar); and (3) Mirid +Whitefly treatment (i.e., plant exposed to mirids in a separated clip cage from the one in which whiteflies were introduced; gray bar). GLM on log-transformed data showed no differences among treatments (*p* > 0.05).

Regardless of the presence of mirids, whiteflies were able to acquire TYLCV in the range of 10^5^–10^6^ copies per ng of whitefly tissue extracted (Figure 3). The presence of mirids inducing defenses for four days in the same leaf neither influenced virus acquisition directly (treatment: Mirid) nor indirectly (treatment: Mirid + Whitefly) (GLM, Chi^2^ = 0.126, df = 2, *p* = 0.939).

## 4. Discussion

The induction of plant defenses has been proposed in recent years as a strategy to reduce pathogen incidence in crops [8,10]. Despite many advances, much is yet to be discovered in relation to the mechanisms that regulate plant defenses and their ecological effects on the crop environment [11]. Here, we studied *D. hesperus*, a representative mirid used for the management of whiteflies in North America [18]. Our hypothesis was that mirid-induced plant defenses could interfere with the process of TYLCV transmission and/or acquisition by whiteflies. We observed a strong induction of plant defenses at the local tissue exposed to mirids. However, this effect did not strongly translate into systemic effects. Exposing plants to mirids for a period of four days had an impact on the host plant choice of *B. tabaci*. Still, this effect did not translate into differences in virus acquisition, transmission, or accumulation. This points to species-specific effects and argues for the careful examination of plant-mediated omnivore effects on each agricultural system.

Omnivorous mirids are known to induce plant defenses through the puncturing of plant tissues [16,23,38]. Here, we corroborated that *D. hesperus* induced plant defenses on the leaf they were exposed to. However, the induction of defenses by mirids is known to differ quantitatively and qualitatively depending on insect species. For example, Pérez-Hedo et al. [22] showed that only *Nesidiocoris tenuis* (Reuter) induced the expression of *ASR1*, the marker gene of the ABA pathway, on tomato plants, while the two other evaluated mirid species (*Macrolophus pygmaeus* Rambur and *Dicyphus marocannus* Wagner) did not. Our results showed no induction of *ASR1* at the local or systemic level, thus suggesting that the induction of defenses by *D. hesperus* resembles that of *D. marocannus*. Most mirid species studied so far (*M. pygmaeus*, *N. tenuis*, *D. marocannus,* and *M. basicornis*) have been also documented to increase JA hormonal levels and upregulate marker genes in the JA pathway in several crop plants [19,20,22,39,40]. Consistent with previous reports, *D. hesperus* induced *PI2C*, a marker gene in the JA pathway locally. In addition, we showed that *D. hesperus* locally induced *PR1a*, a marker gene in the salicylic acid pathway. Altogether, when comparing *D. hesperus* to other mirids, this species was shown to induce plant defenses locally in tomato, mainly through simultaneously activating the SA and JA pathways.

Here, we explored whether confining mirids to a specific leaf could result in Systemic Acquired Resistance (SAR) at a distal point of the plant with potential consequences for plant–virus–vector interactions. Unlike findings on plants treated with SAR elicitors [41,42], *PR1a* was not induced at a distal leaf in our study. A minor difference in *WIPI* expression between a distal leaf in mirid-exposed plants and the local control may indicate that induced responses are systemically expressed. Yet, this systemic effect seems marginal for *D. hesperus*. Zhang et al. [20] also reported that resistance against spider mites induced by a mirid bug was strong at the local leaf of injury but attenuated at the neighboring leaves. Interestingly, the marker gene *SAMT* was induced at both local and systemic leaves of the plants when compared to the levels detected in the local control leaf. This gene encodes for a methyl transferase that increases the levels of methyl salicylate (MeSA) in the plant [9]. MeSA is a volatile compound commonly released upon biotic stress [1,9] but also may function as a signal molecule for SAR activation in distal leaves upon virus infection [7]. Despite evidence for minor systemic effects, our results point mainly to the local induction of defenses by *D. hesperus*.

*Bemisia tabaci* uses visual and chemical cues to select their host plants and strongly responds to color and volatiles emitted by TYLCV-infected plants [43]. Furthermore, *B. tabaci* has been shown to also respond to volatiles elicited by mirids. Specifically, whiteflies showed an innate repellent effect towards tomato plants exposed to *N. tenuis* and also towards specific volatiles induced by mirids [22,25]. Our results indicate that when given a choice, whiteflies avoided settling on plants exposed to mirids, which could be a response towards a specific volatile blend emitted by these plants. Interestingly, in the absence of choice, whiteflies were still settling on mirid-exposed plants. The emission of differential volatile blends by plants upon exposure to omnivores has been confirmed for several mirid species but also for anthocorids and phytoseiid mites [44,45,46]. These volatiles are often terpenoids, Green Leaf Volatiles or MeSA [39,40,44,45], all of which demonstrated to have diverse ecological functions in relation to defense [1]. While we did not perform volatile analysis, a higher expression of *SAMT* was detected in the induced plants, supporting the argument that MeSA could be a relevant volatile induced upon *D. hesperus* exposure to tomato. The induction of volatiles by mirids cascades to other trophic levels, and MeSA may play a relevant role by attracting other natural enemies [44] with further unexplored implications for the success of biological control strategies.

The induction of defenses by *D. hesperus* did not prevent TYLCV transmission to tomato. Apparently, the repellent effect induced by mirids was not sufficient to prevent virus inoculation. TYLCV is a phloem-limited virus transmitted by *B. tabaci* in a highly efficient manner. Only a few viruliferous individuals are required to transmit the virus with an efficiency of 100% in less than 24 h [47]. Furthermore, a single whitefly can retain its ability to transmit the virus for several weeks [48]. Upon virus inoculation, the defenses induced by mirids also did not seem to influence virus replication or movement through the plant. The use of defense elicitors was shown to delay the expression of virus symptoms in tomato and *Nicotiana benthamiana* [12,13]. In contrast, the induction of defenses in mirid-exposed plants did not influence symptom expression or virus accumulation in this study. Although the plants responded to the mirids by eliciting SA plant defenses, *PR1a* was only strongly induced at a local scale. Under these conditions, TYLCV inoculation could effectively occur on a distal leaf.

The induction of plant defenses by mirids did not interfere with virus acquisition by whiteflies either directly or indirectly. In this experimental set-up, infected plants were first exposed to mirids, and then, whiteflies were allowed to acquire TYLCV. Whiteflies acquire TYLCV while feeding from the phloem, a process that entails little cellular damage. During this process, a suite of effectors is secreted with the saliva, resulting in an altered plant defense response [49]. For example, it was shown that feeding by adult whiteflies represses JA-induced defenses in tomato [50]. In this context, the plant defenses induced by mirids were presumably unable to interfere with the process that led to virus acquisition. Also, the presence of mirids in the same clip cage did not directly interfere with the whitefly acquisition of TYLCV. Previously, it was shown that whiteflies can learn to avoid plants colonized by predators that had fed on their offspring [51]. Thus, it is possible that naïve whiteflies were not sensing the risk of predation in our experimental set-up.

While we did not find an effect of defense induction by mirids on TYLCV transmission and accumulation, eliciting plant defenses is an emerging strategy to control plant viruses as alternatives to pesticides [14,52]. Compounds that elicit plant defenses have shown to be effective against a range of biotrophic and necrotrophic pathogens [8,10,11]. Several of these compounds such as SA functional analogs have shown an effect on plant viruses, such as cucumber mosaic virus (CMV) or iris yellows mosaic virus (IYMV) [53,54]. Contrary to our system, these defenses were elicited systemically through the plant. We suggest that this could be a critical aspect to develop virus management strategies. In another example of successful virus management, the induction of plant defenses by defense elicitors in tobacco fields was previously shown to reduce infection rates of tomato spotted wilt virus (TSWV), a virus of the family *Tospoviridae* and order *Bunyavirales* transmitted by thrips [14]. Likewise, controlled transmission assays have shown that the induction of plant defenses by the mirid bug *N. tenuis* can delay the infection of TSWV in sweet pepper (*Capsicum annuum* L.) plants [21]. For begomoviruses, evidence so far shows contradictory results in relation to the use of defense elicitors to control viruses. Defense elicitors delayed the expression of Cassava mosaic disease (CMD) symptoms in *Nicotiana benthamiana* and of TYLCV in tomato under controlled conditions [13,15]. However, treating squash seedlings with acibenzolar-S-methyl under greenhouse conditions did not reduce the incidence of cucurbit leaf crumple virus (CuLCrV) [55]. Given that begomoviruses are phloem-limited, the translocation of defense factors to the phloem may be a critical factor that may determine the success of this approach. Overall, further knowledge on the temporal and spatial effects of defense induction by omnivores and how this influences virus transmission is needed to develop management strategies for insect transmitted plant viruses.

## Figures and Tables

**Table 1:** Marker genes used in this study.

Gene Name	Marker Gene	Pathway	Sequence (5′->3′)	Reference
*Pathogenesis-related protein 1a*	*PR1a*	SA	Fw: TGGTGGTTCATTTCTTGCAACTAC Rv: ATCAATCCGATCCACTTATCATTTTA	[30]
*Salicylic acid carboxyl methyltransferase*	*SAMT*	SA	Fw: TCCCAGAAACATTATACATTGCTGAT Rv: AATGACCTTAACAAGTTCTGATACCACTAA	[31]
*Proteinase Inhibitor IIc*	*PI2c*	JA	Fw: CAGGATGTACGACGTGTTGC Rv: GAGTTTGCAACCCTCTCCTG	[32]
*Wound-induced Proteinase Inhibitor II*	*WIPI*	JA	Fw: GACAAGGTACTAGTAATCAATTATCC Rv: GGGCATATCCCGAACCCAAGA	[33]
*Ethylene responsive factor*	*ERF*	Ethylene	Fw: CGTCCGAGGAAGTGAAACTC Rv: CCGACTCGTAAGTTCCAAGC	[34]
*Abscisic acid Stress Ripening protein 1*	*ASR1*	ABA	Fw: ACACCACCACCACCACCTGT Rv: GTGTTTGTGTGCATGTTCTGGA	[35]
*Actin*	*Actin*	Reference *	Fw: TTAGCACCTTCCAGCAGATGT Rv: AACAGACAGGACACTCGCACT	[36]
*Elongation Factor 1*	*EF1*	Reference **	Fw: GATTGGTGGTATTGGAACTGTC Rv: AGCTTCGTGGTGCATCTC	[35]

* *Actin* served as reference gene for *PR1a*; *SAMT*; *PI2c*; *WIPI*; and *ERF*; ** *EF1* served as reference gene for *ASR1*.

## Data Availability

The raw data supporting the conclusions of this article will be made available by the authors on request.
